# UK healthcare services for people with fibromyalgia: results from two web-based national surveys (the PACFiND study)

**DOI:** 10.1186/s12913-022-08324-4

**Published:** 2022-08-03

**Authors:** Nicky Wilson, Marcus J. Beasley, Catherine Pope, Debra Dulake, Laura J. Moir, Rosemary J. Hollick, Gary J. Macfarlane

**Affiliations:** 1 Departments of Rheumatology and Therapies, King’s College Hospital NHS Foundation Trust, London, UK; 2 Aberdeen Centre for Arthritis and Musculoskeletal Health (Epidemiology Group), University of Aberdeen, Aberdeen, UK; 3 Nuffield Department of Primary Care Health Sciences, University of Oxford, Oxford, UK; 4 Patient Insight Partner, Mansfield, UK

**Keywords:** Fibromyalgia, Health services, UK, Candidacy

## Abstract

**Background:**

The UK’s “Getting It Right First Time” programme recommends that management of people with fibromyalgia should centre on primary care. However, it remains unclear as to how best to organise health systems to deliver services to optimise patient outcomes.

**Aim:**

To profile UK healthcare services for people with fibromyalgia: provision of National Health Services (NHS) and use of non-NHS services by people with fibromyalgia.

**Methods:**

Two online open surveys (A and B) incorporating questions about diagnosis, treatment and management of fibromyalgia and gaps in healthcare services were conducted between 11th September 2019 and 3rd February 2020. These were targeted to NHS healthcare professionals consulting with people with fibromyalgia (Survey A) and people ≥16 years diagnosed with fibromyalgia using non-NHS services to manage their condition (Survey B). Descriptive statistics were used to report quantitative data. Thematic analysis was undertaken for qualitative data.

**Results:**

Survey A received 1701 responses from NHS healthcare professionals across the UK. Survey B received 549 responses from people with fibromyalgia. The results show that NHS services for people with fibromyalgia are highly disparate, with few professionals reporting care pathways in their localities. Diagnosing fibromyalgia is variable among NHS healthcare professionals and education and pharmacotherapy are mainstays of NHS treatment and management. The greatest perceived unmet need in healthcare for people with fibromyalgia is a lack of available services. From the pooled qualitative data, three themes were developed: ‘a troublesome label’, ‘a heavy burden’ and ‘a low priority’. Through the concept of candidacy, these themes provide insight into limited access to healthcare for people with fibromyalgia in the UK.

**Conclusion:**

This study highlights problems across the NHS in service provision and access for people with fibromyalgia, including several issues less commonly discussed; potential bias towards people with self-diagnosed fibromyalgia, challenges facing general practitioners seeking involvement of secondary care services for people with fibromyalgia, and a lack of mental health and multidisciplinary holistic services to support those affected. The need for new models of primary and community care that offer timely diagnosis, interventions to support self-management with access to specialist services if needed, is paramount.

**Supplementary Information:**

The online version contains supplementary material available at 10.1186/s12913-022-08324-4.

## Background

Fibromyalgia is a complex multi-symptom long-term condition that significantly impacts healthcare systems around the world, including in the United Kingdom (UK) [1–4]. Management for people with fibromyalgia, in line with many other long-term conditions, ought to centre on primary care [5–7]. Yet, challenges diagnosing fibromyalgia, its heterogenous symptom profile and frequent coexistence with other diagnoses, and its historical link with rheumatology [8–11] mean that people with fibromyalgia commonly interact with healthcare professionals in a range of services and settings.

Treatment guidelines and care recommendations for people with fibromyalgia incorporate individually tailored pharmacological and non-pharmacological interventions to support self-management and address patient symptoms [5, 12, 13]. However, treatment patterns show inconsistent use of evidence-based interventions [14–18] and patients with fibromyalgia express low levels of satisfaction with the healthcare they receive [19]. In the UK, where 85% of healthcare is provided free at the point of delivery, people with fibromyalgia report difficulty accessing services, limited support from healthcare professionals to manage their condition and difficult patient-provider relations [20–22].

Integration between healthcare services is one way to improve patient satisfaction and increase access to care [23]. However, the way in which to organise health systems to deliver services that optimise outcomes in patients with fibromyalgia is unclear and has been highlighted as a knowledge gap in recent EULAR guidelines for the management of fibromyalgia [13]. A systematic review by Doebl et al. [19] failed to identify any evidence-based model of care for people with fibromyalgia that traversed the entire healthcare system.

In response to uncertainty about how best to organise health services for people with fibromyalgia, a large programme of research called PACFiND - PAtient-centred Care for Fibromyalgia: New pathway Design - has been launched. PACFiND is a suite of studies that aims to collect information from patients and healthcare professionals about UK healthcare services for people with fibromyalgia. The programme includes analysis of routinely collected data to enable mapping of patient healthcare journeys, the identification, through in-depth case studies across the UK, of better or best care (informed by evidence and the patient voice) and cost-benefit analyses of different models to guide development and co-design of new pathways of care for people with fibromyalgia. This manuscript is one component of PACFiND and seeks to profile healthcare for people with fibromyalgia in the UK: provision of NHS services and use of non-NHS services by people with fibromyalgia. The study objectives were to identify:

- Which healthcare professionals diagnose fibromyalgia and the tools used to support diagnosis.
- Which healthcare professionals treat and/or manage fibromyalgia and the treatments provided.
- Non-NHS treatments and services people with fibromyalgia access to manage their condition.
- Gaps in current healthcare services for people with fibromyalgia.
- Possible case study sites for further research into service provision.

## Methods

Two online open surveys (A and B) consisting of web-based questionnaires were conducted using Research Electronic Data Capture (REDCap) software (https://redcap.abdn.ac.uk). Both surveys were conducted prior to the start of the COVID-19 pandemic; survey A between 11th September 2019 and 5th January 2020, and survey B between 13th January and 3rd February 2020. The target population for survey A was NHS healthcare professionals consulting with people with fibromyalgia or with signs and symptoms suggestive of fibromyalgia, within the last 2 years. Survey A was designed to gather demographic data about respondents, information about diagnosing fibromyalgia, its treatment and management, and perceived gaps (if any) in the provision of services for people with fibromyalgia. Survey B was targeted at people aged 16 years or older living in the UK with a diagnosis of fibromyalgia and using non-NHS services to help them self-manage their condition. Survey B questions were grouped under three subheadings: demographic information; use of non-NHS services (including type of treatment/service, frequency, reasons for and experiences of access); and other comments. To help identify potential case study sites, participants were invited to enter the address / postcode of their NHS service or practice (survey A) and the non-NHS organisation or provider they accessed to help manage their condition (survey B).

The questionnaires were developed in close consultation with healthcare professionals, patient research partners and people with fibromyalgia, and included a mix of closed and open-ended questions. Survey A was registered in Scotland as a service evaluation; survey B was approved by the University of Aberdeen School of Medicine, Medical Sciences and Nutrition Ethics Review Board (CERB/2019/11/1805). Participants provided informed consent prior to accessing the surveys.

An invitation to participate in survey A, along with a link to the questionnaire, was distributed in primary care by contacting Clinical Commissioning Groups in England and the Scottish Primary Care Research Network in Scotland. Contacts were asked to include the information in newsletters and mailing lists. In Wales and Northern Ireland, email invitations with the survey link were sent directly to general practices. To distribute the survey in secondary care, NHS Trusts in England, Health Boards in Scotland, and Health & Social Care Trusts in Northern Ireland were contacted and asked to share an invitation to the survey. Additionally, the survey was circulated through professional networks and organisations. Survey B was advertised via the websites and social media channels of Versus Arthritis and Fibromyalgia Action UK and members of the PACFiND programme patient and public involvement group. Both surveys were publicised through the twitter feeds of the PACFiND project and its investigators, and on the University of Aberdeen-hosted PACFiND website.

### Data analysis

Data were exported from REDCap to Microsoft Excel and Stata/SE15.1 for cleaning and analysis. Complete and incomplete questionnaires with an individual date and timestamp were included in the analysis provided i) informed consent to participate was recorded and ii) responses contributed data about the organisation, delivery and use of care for and by people with fibromyalgia. Quantitative data were analysed descriptively; summarised by the number of respondents answering a question in each category and expressed as a percentage of the total number of people answering that question. Qualitative data from responses to open ended questions were imported into NVivo 12 (QRS International), a computer software package, and coded to capture the essence of the text. Codes were organised and grouped into categories, and subthemes and themes were developed using the constant comparative method [24, 25]. Analysis of the free text data was both inductive and deductive. Survey methods are reported according to the Checklist for Reporting Results of Internet E-Surveys (CHERRIES) statement [26].

## Results

Survey A received 1701 responses, and survey B received 549 responses providing data about the organisation, delivery and use of UK healthcare services for, and by people with fibromyalgia. Of the 1701 records in survey A, 1122 (66.0%) respondents’ primary role was in England, 329 (19.3%) in Scotland, 190 (11.2%) in Wales, 53 (3.1%) in Northern Ireland, and for 7 (0.4%), no location was given. The healthcare setting for 701 (41.2%) respondents was general practice. Other healthcare settings were acute hospital (559, 32.9%), community hospital (264, 15.5%) and primary healthcare centres (137, 8.1%). Another setting or no setting was stated for 40 (2.4%) respondents. General practitioner was the main job for 642 (37.7%) respondents, while hospital doctors (194, 11.4%) included 111 rheumatologists, 53 physicians in pain medicine and 30 categorised as other, such as doctors working in emergency medicine and anaesthetics. Allied health professionals, nurses and mental health professionals (mental health practitioners, psychiatrists and psychologists) made up 26.1, 13.8 and 6.0% of the total records respectively. Other respondents (*n* = 84, 4.9%) included pharmacists, service managers, occupational health advisors and midwives. One respondent did not provide information about their primary role. Of the 549 records in Survey B, 335 (61.0%) participants lived in England, 98 (17.9%) in Scotland, 107 (19.5%) in Wales and nine (1.6%) in Northern Ireland.

Our findings (both quantitative and qualitative) are presented under the headings: ‘diagnosis’; ‘treatment and management of people with fibromyalgia’; and ‘gaps in healthcare services’. Quantitative data are shown in Tables 1, 2 and 3, while Table 4 contains examples of qualitative data underpinning key themes.

**Table 1:** Healthcare professionals diagnosing fibromyalgia and use of diagnostic tools

Main job	Do you diagnose fibromyalgia?		Tools used for diagnosis					
	No, n (n/N %)	Yes, n (n/N %)	Clinical opinion, n (n/N %)	Tender point exam, n (n/N %)	WPI, n (n/N %)	SS Scale, n (n/N %)	Other, n (n/N %)	No tool, n (n/N %)
	*N* = 979	*N* = 717	*N* = the number of the professional group who state they make the diagnosis					
General Practitioner	194 (30.3)	446 (69.7)	396 (88.8)	234 (52.5)	112 (25.1)	85 (19.1)	21 (4.7)	2 (0.4)
Hospital doctor								
Pain Medicine	9 (17.0)	44 (83.0)	37 (84.1)	15 (34.1)	25 (56.8)	25 (56.8)	7 (15.9)	0 (0.0)
Rheumatology	0 (0.0)	111 (100.0)	106 (95.5)	88 (79.3)	48 (43.2)	25 (22.5)	6 (5.4)	0 (0.0)
Other	25 (83.3)	5 (16.7)	4 (80)	4 (80)	3 (60.0)	2 (40.0)	0 (0.0)	0 (0.0)
Psychiatrist	3 (75.0)	1 (25.0)	1 (100)	0 (0.0)	0 (0.0)	0 (0.0)	0 (0.0)	0 (0.0)
Nurse								
General Practice	41 (82.0)	9 (18.0)	7 (77.8)	4 (44.4)	5 (55.6)	3 (33.3)	0 (0.0)	1 (11.1)
Pain Medicine	38 (73.1)	14 (26.9)	12 (85.7)	8 (57.1)	11 (78.6)	10 (71.4)	1 (7.1)	0 (0.0)
Rheumatology	39 (72.2)	15 (27.8)	12 (80.0)	12 (80.0)	7 (46.7)	3 (20.0)	0 (0.0)	0 (0.0)
Other	77 (98.7)	1 (1.3)	1 (100.0)	1 (100.0)	1 (100.0)	1 (100.0)	0 (0.0)	0 (0.0)
Allied Health Professionals	384 (86.5)	60 (13.5)	51 (85.0)	20 (33.3)	40 (66.7)	32 (53.3)	8 (13.3)	0 (0.0)
Mental Health Professionals	93 (95.9)	4 (4.1)	3 (75.0)	0 (0.0)	0 (0.0)	0 (0.0)	2 (50.0)	0 (0.0)
Other	76 (90.5)	7 (8.2)	7 (100)	1 (14.3)	4 (57.1)	3 (42.9)	1 (14.3)	0 (0.0)
**Total**	979 (57.7)	717 (42.3)	637/717 (88.8)	387/717 (54.0)	256/717 (35.7)	189/717 (26.4)	46/717 (6.4)	3/717 (0.4)

*Abbreviations*: *WPI* Widespread Pain Index, *SS Scale* Symptom Severity Scale

**Table 2:** Provision of treatment/management to people with fibromyalgia by NHS healthcare professionals

Main job	Treatment/ management?		Education & / or information leaflet n (n/N %)	Medicines prescription &/or OTC n (n/N %)	Psychological therapies n (n/N %)	Structured exercise n (n/N %)	Multicomponent programme n (n/N %)	Non-pharmacological (Other) n (n/N %)
	No, n (n/N %)	Yes, n (n/N %)						
	*N* = 309	*N* = 1381	*N* = the number of the professional group who provide treatment/management					
General Practitioner	48 (7.5)	588 (92.5)	547 (93.0)	533 (90.6)	191 (32.5)	121 (20.6)	93 (15.8)	63 (10.7)
Hospital doctor								
Pain Medicine	1 (1.9)	52 (98.1)	46 (88.5)	47 (90.4)	23 (44.2)	18 (34.6)	33 (63.5)	15 (28.8)
Rheumatology	15 (13.5)	96 (86.5)	94 (97.9)	77 (80.2)	21 (21.9)	34 (35.4)	22 (22.9)	7 (7.3)
Other	22 (73.3)	8 (26.7)	6 (75.0)	3 (37.5)	2 (25.0)	5 (62.5)	2 (25.0)	0 (0.0)
Psychiatrist	1 (25.0)	3 (75.0)	3 (100.0)	1 (33.3)	2 (66.7)	0 (0.0)	1 (33.3)	0 (0.0)
Nurse								
General Practice	28 (57.1)	21 (42.9)	17 (81.0)	17 (81.0)	7 (33.3)	4 (19.0)	1 (4.8)	0 (0.0)
Pain Medicine	2 (3.8)	50 (96.2)	49 (98.0)	44 (88.0)	32 (64.0)	21 (42.0)	39 (78.0)	15 (30.0)
Rheumatology	14 (26.4)	39 (73.6)	39 (100.0)	28 (71.8)	8 (20.5)	8 (20.5)	11 (28.2)	5 (12.8)
Other	59 (76.6)	19 (24.7)	9 (47.4)	12 (63.2)	5 (26.3)	1 (5.3)	2 (10.5)	2 (10.5)
Allied Health Professionals								
Occupational Therapist	8 (7.9)	93 (92.1)	88 (94.6)	3 (3.2)	28 (30.1)	15 (16.1)	34 (36.6)	35 (37.6)
Physiotherapist	28 (9.2)	276 (90.8)	264 (95.7)	53 (19.2)	70 (25.4)	233 (84.4)	137 (49.6)	66 (23.9)
Other	13 (33.3)	26 (66.7)	16 (61.5)	3 (11.5)	2 (7.7)	3 (11.5)	2 (7.7)	21 (80.8)
Mental Health Professionals								
Mental health practitioner	20 (54.1)	17 (45.9)	11 (64.7)	0 (0.0)	15 (88.2)	0 (0.0)	1 (5.9)	1 (5.9)
Psychologist	7 (11.7)	53 (88.3)	45 (84.9)	3 (5.7)	51 (96.2)	4 (7.5)	36 (67.9)	1 (1.9)
Other	43 (51.8)	40 (47.6)	31 (77.5)	22 (55.0)	9 (22.5)	11 (27.5)	12 (30.0)	12 (30.0)
**Total**	309 (18.3)	1381 (81.7)	1265/1381 (91.7)	846/1381 (61.3)	466/1381 (33.7)	478/1381 (34.6)	425/1381 (30.8)	243/1381 (17.6)

**Table 3:** Healthcare professionals’ perceptions of gaps in healthcare services for people with fibromyalgia

Are there gaps in your local healthcare services?	Number of people identifying this as being the most important unmet need, n (n/N %)	Number of people identifying this as an important unmet need (including those identifying it as most important), n (n/N %)
	*N* = 1601	*N* = 1601
Yes, lack of available services	499 (31.2)	892 (55.7)
Yes, lack of healthcare professionals’ knowledge/skills	252 (15.7)	652 (40.7)
Yes, long wait times to appointments	113 (7.1)	633 (39.5)
Yes, funding issues	32 (2.0)	330 (20.6)
Yes, lack of time during appointments	31 (1.9)	433 (27.1)
Yes, limited communication/coordination between providers	23 (1.4)	266 (16.6)
Yes, restrictive service delivery policies	23 (1.4)	255 (15.9)
Yes, continuity of relations between provider and patient	19 (1.2)	229 (14.3)
Yes, limited transport availability	10 (0.6)	192 (12.0)
Yes, lack of access to shared medical records	3 (0.2)	156 (9.7)
Yes, other important unmet need	36 (2.3)	105 (6.6)
Yes, no important unmet need given	104 (6.5)	
No	182 (11.4)	
Don’t know	274 (17.1)	
**Total**	1601 (100.0)

**Table 4:** Themes, subthemes and example comments

Theme	Sub-theme	Example comments
Diagnosis: A troublesome label	Diagnostic uncertainty and delay	“The patients that I see generally have physical and psychiatric co morbidity. They usually have a diagnosis of fibromyalgia amongst a number of other somatoform disorders and/or mood disorder and/or chronic syndrome[s], and it is unclear whether fibromyalgia is actually a separate diagnosis.” (A-295; Psychiatrist, Greater London) “The difficulty is confidently making the diagnosis in primary care - 1. you may miss a life-threatening illness eg Cancer …, Polymyalgia 2. if the diagnosis is technically correct some [patients] seem to spend the rest of their lives validating it and forgetting to live.” (A-1288; GP, Wales) “I think all clinicians need a better understanding of FMS [Fibromyalgia Syndrome] and associated functional somatic disorders to make [a] confident diagnosis of FMS like we do with IBS [Irritable Bowel Syndrome] and stop referring them [people with fibromyalgia] round in circles from one speciality to the next - this would be better for patients and save [the] NHS a fortune.” (A-52; GP, West Midlands) “[…] There seems to be a lack of diagnostic confidence. The long wait for a rheumatology appointment and uncertainty during that time engenders significant anxiety that does not help patients.” (A-142; Rheumatologist, Scotland) “[A] lack of skill or confidence in making a diagnosis in primary care means people are not given early advice or information about how to manage their condition.” (A-669; Psychologist, South West England)
	The indelible nature of fibromyalgia	“‘Fibro’ has now become a common label for a whole cohort of young people presenting often with a self-diagnosis and a negative mindset about their future capabilities and sadly a negative engagement with non-drug options.” (A-1669; GP, South East England) “Dr Google also causes self-diagnosis which unfortunately makes it challenging as it only needs one healthcare professional to make an innocent comment of agreement, and this is then in stone.” (A-1392; Physiotherapist, Wales) “[A] lack of engagement by patients; some tend to ‘wallow’ in diagnosis and [it is] difficult to change their mindset.” (A-1139; GP, Scotland) “Once a diagnosis of fibromyalgia is given, other professionals ‘blame’ all the patient’s ills on this diagnosis and stop being open minded and vigilant. I think fibromyalgia should be used selectively and carefully, so that it enhances a patient’s experience [….].” (A-986; Physiotherapist, West Midlands) “The elephant in the room is where a diagnosis is linked to staying on benefits which means this a psychosocial illness, which I would call a posture rather than an illness. Developing pathways of care may embed an illness model which does not really help patients long term.” (A-689; GP, South East England) “Fibromyalgia is a ‘diagnostic dustbin’ that people are put in, or put themselves in. Because it is a chronic illness with no treatment … I avoid it. […] How can anyone ‘prove’ this diagnosis/label, and why do so? It is often used to ‘dump’ patients that doctors can’t be bothered to try to assess any more.” (A-616; GP, South East England)
	An empty diagnosis	“Poor education on [the] condition at the time of diagnosis.” (A-1563; Physiotherapist, Greater London) “On diagnosis of my fibromyalgia, I received no advice or help. I was just told that that is what I had!” (B-363, England) “We have limited capacity to see fibromyalgia patients when we are already stretched seeing [our] inflammatory cohort. This should be a diagnosis that can be made in the community […]. We are unable to offer more than a diagnosis confirmation [and] pointers to self-management at rheum[atology] outpatients. We make some management suggestions but do not offer ongoing care as no staffing.” (A-142; Rheumatologist, Scotland)
Treatment and management: A heavy burden	No route map	“A lot of my patients have inflammatory arthritis e.g. Rheumatoid Arthritis. I find that many GPs refuse to engage with patients who are under our care. There is a lack of a local pathway that gives all providers responsibility.” (A-1450; Nurse in rheumatology, Greater London) “[The] CCG have not streamlined management of persistent pain effectively; two pain management pathways [are] present within [the] local area; one that is very effective, one that isn’t. Patients are unfortunately the ones who don’t benefit from this.” (A-386; Physiotherapist, South East England) “No consistent package across Wales - some health boards rheumatologists refuse to see patients with a primary diagnosis of fibromyalgia, others do see but little resource in house, outsourcing and no central coordination within the health care team mean[s] that patient[s] gets lost in a myriad of suggestions as opposed to empowerment and oversight and then patients seek multiple opinions as [they are] unsure what to expect.” (A-107; Rheumatologist, Wales) “Effective management of fibromyalgia often requires services working collaboratively e.g. rheumatology, pain clinic, physiotherapy, psychology and mental health services. These services are often not under one roof and often are in separate trusts which hinders collaborative working.” (A-309; Psychologist, West Midlands) “The biggest issue for the care given to this patient group, I find, is that their condition is managed in a ‘disjointed’ ‘symptom by symptom’ approach, which increases their waiting times and [reduces] their quality of life in the meantime.” (A- 264, Dietitian, East Midlands)
	An unwelcome condition	“No one discipline wants to take the ownership of care for fibromyalgia patients - they are viewed as a burden.” (A-1480; Physiotherapist, North West England) “We have recently been advised that our community MSK service and rheumatology at [Name] hospital do not accept referrals for patient[s] with fibromyalgia as their primary diagnosis, even …to screen them for [the] pain clinic. They are rejected for [the] GP to manage.” (A-293; Physiotherapist, East of England) “No interest from secondary care specialist so where patients are not improving or have unusual symptoms, we struggle to obtain support/secondary care opinion.” (A-1130; GP, Scotland) “I’ve been told by doctors [that] physiotherapy would help me. Then told …they [the doctors] can’t refer me, but they would if I had a different diagnosis.” (B-542, Scotland) “I find its always handed over with an ‘eye roll ‘.” (A-1228; Nurse, Scotland) “We see patients in rheumatology where the needs of the patients would be best met in a service with dedicated psychology. The NHS is still separating mental and physical health where fibromyalgia needs a combined approach. Our hospital won’t pay for a psychologist so we have to refer on to [the] pain team which produces a disjointed pathway. Pain team only accept referrals for patients who have psychological issues related to pain, whereas what we see with fibromyalgia patients, are people who have long standing mental health issues that often result or contribute to fibro symptoms. So [the] pain team can reject the referrals, which leaves us with almost no options for specific mental health support.” (A-1069; Occupational Therapist, Yorkshire and the Humber)
	Unhelpful pharma and the cost of keeping going	“Services like specialist care or pain clinic[s] seem to just be giving high dose combination addictive non-evidence based long term analgesia, which is not appropriate.” (A-574; GP, Yorkshire and the Humber) “I work for a community pain service ...GP[s] tend to overuse medication even if it is providing limited benefit.” (A-1496, Nurse in pain medicine, South East England) “[I am] fortunate to have some private healthcare insurance that was taken out many years before diagnosis [of fibromyalgia]. [I] get some help towards [the] expense of acupuncture but [it is] still very expensive …to prevent being unable to function / work some hours.” (B-26, England).
Service gaps: A low priority	A barren landscape	“My consultant asked [my] GP to refer me to [a] ‘specialist fibromyalgia clinic’. My GP apologised and [said] there ‘was no such clinic’.” (B-368, England) “The numbers of FM (fibromyalgia) pts [patients] have quadrupled over the last 4 years. We only have the capacity to run 1 group a week. Also there is very little in the way of community services - this automatically medicalises the treatment once the group is in the hospital.” (A-279; Occupational Therapist, South East England) “Not all patients get access to a holistic pain management programme, and sometimes just get offered medication in pain clinics - I think ALL patients should be supported holistically, and repeatedly if they don’t engage the first time round.” (sic) (A-813; GP, Yorkshire and the Humber) “Funding constraints approx. a year ago led to fibromyalgia generally being dealt with by GPs (no further secondary care nor multi-component programmes offered).” (A-1698 Yoga Therapy Teacher, South West England) “We have very limited options for referral - including exercise referral and the EPP [Expert Patient Programme] which is run through the voluntary sector. There is a huge need for additional patient education and support in the community, to enable patients to seek help in self-management.” (A-1364; GP, Wales)
	Nothing long term	“Due to limited resources, there is little scope for long term follow up/check-ups. Other long-term conditions are more likely to have an annual review. Patients with FMS attending our service are given appropriate information and signposted to community resources when they are discharged but may struggle to implement/access due to multifactorial reasons.” (A-226; Physiotherapist, Scotland) “Services aren’t able to be responsive to acute flare ups and have limited capacity to provide ongoing support whilst individuals develop their self-management skills.” (A-208; Physiotherapist, South East England) “NHS care is great for short term conditions, but it has a long way to go with managing long term conditions, especially pain. Fibro patients often need things like long term physiotherapy and most clinics only provide 6 sessions which barely scratches the surface with such a complex disease.” (B-17, England)
	[Un]knowledgeable professionals	“It appears many clinicians have a poor understanding of fibromyalgia. Sadly, it is not uncommon for patients with fibromyalgia to report to me that there are still professionals they encounter who believe their symptoms represent some sort of medically unexplained or ‘functional disorder’ and that their symptoms are somehow less valid than other, better understood disorders.” (A-391; Psychologist, Yorkshire and the Humber) “[There is] little training provided to Orthoptists who often see patients with fibromyalgia related eye symptoms.” (A-402; Orthoptist, South West England) “The unmet need is an educational one of professionals. […] This includes GPs.” (A-680; GP, North East England) “This survey has made me reflect, am I doing enough for the people I see in clinic? I don’t know what services are available in my area. I don’t talk to them [people with fibromyalgia] about holistic things such as sleep hygiene, pain management, pacing.” (A-1672; Podiatrist, Yorkshire and the Humber)

### Diagnosis

Of 1697 respondents who answered the question “Do you diagnose fibromyalgia?”, 717 (42.3%) reported they did (Table 1). Of those, the majority diagnose adults only (86.6%) or both adults and adolescents (12.7%). By speciality, the greatest proportion of professionals diagnosing fibromyalgia were rheumatologists (100.0%), followed by physicians in pain medicine (83.0%) and general practitioners (69.7%). Of the non-medical professionals, 20.1% of nurses, 13.5% of allied health professionals and 4.1% of mental health practitioners and psychologists stated they diagnose fibromyalgia. Tools to support fulfilment of fibromyalgia diagnostic criteria were used inconsistently by clinicians diagnosing fibromyalgia. Just over a quarter of NHS healthcare professionals (26.0%) stated they rely on clinical opinion alone when diagnosing fibromyalgia, 13.0% supplement clinical opinion with the Widespread Pain Index (WPI) and Symptom Severity (SS) Scale, while 54.0% report using the tender point examination.

Of the 980 respondents who indicated they do not diagnose fibromyalgia, 561 (57.2%) reported referring to other providers for diagnosis, most commonly a rheumatologist (73.6%), a general practitioner (43.0%) and a physician in pain medicine (22.8%). Analysis of free text data about diagnosis of fibromyalgia generated the theme ‘A troublesome label’.

### Theme: A troublesome label

For many NHS professionals the process of diagnosing fibromyalgia focussed on recognising a custom symptom profile and excluding organic disease. Some clinicians reported using published diagnostic frameworks, such as the ACTTION-APS Pain Taxonomy (AAPT) and the 2010 / 2016 American College of Rheumatology criteria [27–29], while others drew on locally developed guidance and checklists produced by national charities. Notwithstanding this, several respondents considered contemporary diagnostic frameworks for fibromyalgia unsatisfactory, with one general practitioner highlighting the challenges associated with diagnosing fibromyalgia in practice.

As there is no objective confirmatory test for fibromyalgia, patients these days tend to self-diagnose and present with the typical history. The tender point examination is pretty unhelpful once a patient has self-diagnosed with fibromyalgia. (A-1148; General Practitioner (GP), Scotland)**.**

#### Sub-theme: diagnostic uncertainty and delay

Self-diagnosis, misdiagnosis and over-diagnosis, alongside perceptions of rising numbers of people with fibromyalgia concerned NHS healthcare professionals, although respondents’ understanding of the condition varied. Fibromyalgia’s fuzzy boundary led several healthcare professionals to query its distinctiveness in the presence of other syndromes and diagnoses, while others typified the condition as a mind-body illness, a psychological disorder, a modern day ‘inflammatory’ condition, synonymous with chronic pain, and, in the account below, reflective of individual character traits.

I dispute the actual diagnosis exists. I suspect they [people with fibromyalgia] have an undiagnosed mental illness or are just plain lazy and need to get a job and get on with life. (A-640; GP, South East England).

Low levels of skill and confidence to diagnose fibromyalgia among healthcare professionals, in particular general practitioners, was reported, along with missed opportunities to instigate early self-management because of delayed diagnosis. Some healthcare professionals highlighted a need for greater understanding about fibromyalgia to prevent patients from being referred around in circles. Several general practitioners expressed a lack of confidence to diagnose fibromyalgia, underpinned by fear of missing serious pathology or misattribution of a diagnosis with significant impact. Patients who self-diagnosed fibromyalgia seemed especially challenging to healthcare professionals. Comments from some NHS professionals hinted at associations between self-diagnosis and a propensity to ‘opt out’ of society and seek social benefits. For one hospital doctor the need for an easy and convenient test to differentiate between people with “real fibromyalgia and those who twist the system to diagnose themselves as fibromyalgia” was paramount (A-1462, Physician in pain medicine, West Midlands).

The power of diagnosis lies in its potential to explain things previously puzzling and delineate the path ahead [30]. While a few healthcare professionals reported making a ‘positive’ diagnosis in people with fibromyalgia - seeing it as an opportunity to pause, reset and pursue appropriate management strategies, others considered a fibromyalgia diagnosis of limited utility. Similar to findings from a study by Rasmussen [31], wherein general practitioners in Norway were reluctant to confer a diagnosis of fibromyalgia because of anticipated unhelpful consequences, a couple of the general practitioners in our study stated they avoided diagnosing fibromyalgia, based on perceptions about the label’s poor explanatory and prognostic value and its embodiment of long-term disability. One general practitioner commented that he hated making a diagnosis of fibromyalgia as it led to “a life of analgesia and general comorbidity” (A-1226, GP, Scotland), while in relation to care following diagnosis, an occupational therapist expressed apprehension about the label’s potential to negatively influence therapeutic opportunities.

[An] other concern is … [the] negative reaction [of staff] to the diagnosis and concern that [the] patient will not make any positive progress. (A-1238; Occupational Therapist, Scotland).

#### Sub-theme: the indelible nature of fibromyalgia

Perceptions about the indelibility of the fibromyalgia label additionally caused unease. Once given, a label of fibromyalgia was viewed by some healthcare professionals as hard to move past, leaving patients stuck in a cycle of unhelpful illness behaviours with limited opportunity for recovery. For a few respondents the continued preponderance of the fibromyalgia label risked diagnostic overshadowing, described by Iezzoni [32] as the ‘erroneous attribution of all new symptoms to an underlying health condition’ (p.2093). One person living with fibromyalgia commented that since being diagnosed with the illness, any issue they developed was “… almost always … blamed on my fibro” (B-246). The potential for diagnostic overshadowing becomes understandable in the light of a comment from one general practitioner diagnosing and treating patients with fibromyalgia, although it is unclear whether the ‘register’ mentioned in the account below is real or metaphorical:

[After diagnosis and treatment of patients with fibromyalgia] add to [the] register of patients to prevent prescribing and referral. (A-707, GP, South West England).

The diagnostic turbulence ([33], p2) evident in the accounts above, offers some insight into the number of healthcare professionals referring to other healthcare professionals to make or confirm a diagnosis of fibromyalgia, notably rheumatologists. In a few localities, rheumatology services diagnosed fibromyalgia routinely, in line with local pathways and or individual clinician referral behaviours: one general practitioner stated that fibromyalgia should only be diagnosed in secondary care as it could be “a devastating diagnosis of a severe chronic condition” (A-1351; GP Wales). While these arrangements were satisfactory for some, the disadvantages of these referral routes were highlighted by professionals and people with fibromyalgia, such as rising anxiety for patients during the often-long wait for a specialist appointment and a lack of a clear post-diagnosis management plan, influenced by a ‘diagnose and discharge model’ in outpatient secondary care services.

We can usually only offer a diagnostic opinion [for people with symptoms suggestive of fibromyalgia] (if possible, with management advice given at that appointment). (A-120; Rheumatologist, South West England).

My most recent patient diagnosed [with fibromyalgia] by the rheumatology department … was simply given the diagnosis and discharged with no follow up and no management plan. (A-1110; GP, Scotland).

#### Sub-theme: an empty diagnosis

The emptiness of the fibromyalgia diagnosis in the account above, at the heart of which is a failure to expand patient understanding about their illness experience and future management, has been recognised [34, 35]. While most NHS healthcare professionals providing treatment and management to people with fibromyalgia stated they offer information and education (see Table 2), some respondents commented on a lack of a comprehensive resource available for those affected. One nurse suggested fibromyalgia specific education post diagnosis, similar to DESMOND (Diabetes Education and Self-Management for Ongoing and Newly Diagnosed) for people with diabetes, while a general practitioner, themselves living with fibromyalgia, emphasised the vital link between patient education and effective self-management.

[There is a] lack of education provided to patients. I am so lucky that I have a reasonable understanding of my body [and] what I need to do to manage this [my fibromyalgia]. … many patients don’t [and] so are unmotivated to help themselves. This is essential in managing fibro[myalgia]. (A-617; GP, Greater London).

### Treatment & management of people with fibromyalgia

Out of the 1690 NHS healthcare professionals who answered the question “Do you personally provide treatment or management for people with fibromyalgia?”, 1381 (81.7%) said they did (see Table 2). Of the 1619 who answered the question “Do you refer patients with fibromyalgia to other providers for treatment?” 1147 (70.8%) answered yes. Commonly, referrals were to physiotherapists (54.1%), physicians in pain medicine (42.9%) and rheumatologists (40.5%). Fewer referrals for treatment were made to a psychologist (28.5%), a mental health practitioner (26.2%), or an occupational therapist (25.5%).

After education and information, the treatment most frequently offered to people with fibromyalgia was a prescription medicine or a recommendation for an over-the-counter medicine (61.3% of respondents). The most common non-NHS treatments accessed by people with fibromyalgia were massage therapy (40.1%), mindfulness (38.8%) and support groups (38.1%), although many respondents reported using multiple interventions to manage their condition. Approximately one third of NHS healthcare professionals stated they provide non-pharmacological interventions such as structured exercise (34.6%), psychological therapies (33.7%), and multicomponent programmes (30.8%), with over half of the professionals delivering multicomponent programmes working in NHS pain services. Of those supplying information about their multicomponent programmes (369/425), 248 (67.2%) offered all three key interventions - education and advice, exercise and psychological therapies. The most popular delivery model for multicomponent programmes was one session weekly for 6 weeks.

Participant responses highlighted wide disparity in service provision. A few NHS professionals stated they offered or had access to specialist fibromyalgia services or fibromyalgia-specific programmes and education groups, mostly based in secondary care. Others reported no specialised or specific service provision for patients with fibromyalgia but made use of mainstream therapy services or pain management programmes. However, respondents’ views about the appropriateness and effectiveness of care delivered in these settings varied. Reports of services embedded in or linked to general practice to support people with fibromyalgia were few and far between, although one clinical pharmacist in South West England stated they offered a monthly fibromyalgia support group across their primary care network. Typically, participants across both surveys highlighted inadequate timely treatment and management opportunities for people with fibromyalgia leading to the theme ‘A heavy burden’.

### Theme: A heavy burden

Pathways of care can promote equitable services for people with specific health conditions [36]. However, only a few healthcare professionals reported having a local pathway of care for patients with fibromyalgia. In one or two localities, care processes and interventions were organised and delivered solely in primary care, while in other areas, care provision spanned primary, secondary, and tertiary services. Inadequate care elements, limited communication between professional teams about the care of people with fibromyalgia, and a lack of co-ordinated care processes led to symptom-by-symptom management, an array of different referral routes (each with inbuilt delays), and “a postcode lottery for the quality of services available” (A-1483; Nurse in rheumatology, Yorkshire and the Humber). One rheumatologist explained the impact of this shortfall.

In common with many other locations, we have significant discontinuity in primary care and fragmentation of service provision which can result in confusion for the patient and unnecessary duplication of services. Patients frequently cycle through multiple services repeatedly and are at risk of medicalisation. (A-07; Rheumatologist, South East England).

#### Sub-theme: no route map

Along with the lack of local care pathways, many NHS professionals recounted experiences of substantial difficulties accessing recommended interventions for patients with fibromyalgia. Accounts from practitioners working in primary care highlighted the struggle to have referrals for people with fibromyalgia accepted by physical and mental health services, although some professionals working in secondary care also found access to ‘in house’ interventions problematic.

We have a large patient population within our practice with fibromyalgia and no secondary [care] based consultant will accept a referral and physio success is limited. Patients feel unsupported and left with no secondary care input. (A-176; Nurse in General Practice, Wales).

We have been refused access to the self-management programme run for FMS [Fibromyalgia Syndrome] patients by our Trust’s pain clinic… the service was overwhelmed. (A-1491; Rheumatologist, South West England).

One general practitioner summed up their experience of seeking care for patients with fibromyalgia – “‘not’ rheumatology, ‘not’ pain clinic, ‘not’ psychological therapies, ‘not’ physical therapies” (A-590; GP, North West England), while another described the frustration accompanying referral efforts.

Secondary care seems to fail to understand that 99% of fibromyalgia is managed in primary care. On the whole we [general practitioners] can manage it well and have a good level of understanding. It is no longer a ‘diagnosis of exclusion’ but something we actively engage with. On the rare occasions we feel out of our depth or have patients with complex symptoms that we struggle with, we need a service that will accept a referral, do a proper assessment, and provide a service to that patient. At present services simply pass the buck or …reject referrals. (A-1302; GP, Wales).

#### Sub-theme: an unwelcome condition

Underpinning difficulties accessing care were a range of influences: perceptions about the burden associated with caring for people with fibromyalgia were mentioned frequently in comments from healthcare professionals; provider experiences of outcomes, which one GP described as “rarely good” (A-716; GP, South West England); local and regional service policies; and the separation of mind from body that impacts responsibility for healthcare provision.

We [physiotherapy] do not accept referrals for fibromyalgia. We signpost to self-help management strategies in line with [local health board] policy (A-1424; Physiotherapist, Wales).

Psychology will not see patients with FM [fibromyalgia] without an additional mental health diagnosis (A-1141; GP, Scotland).

These, and other comments, give insight into the contested space occupied by people with fibromyalgia in the NHS. Some general practitioners and people with fibromyalgia viewed the rhythm and landscape of general practice as incompatible with caring for people with fibromyalgia, drawing attention to the challenges associated with managing fibromyalgia’s inherent complexity within traditional 10-minute general practice appointments.

General practice is not the ideal place for these patients to be managed. It results in their condition worsening, in addition to inappropriate polypharmacy. (A-1361; GP, Wales).

In contrast, other professionals, notably those working in hospital-based services (but also some from general practice), purported that care was best when positioned in less medical settings, albeit in the context of adequate available resource within general practice and the community.

This should be a diagnosis that can be made in the community and there should be better community-based support - physio, exercise classes, psychology input available to help GPs and their patients with fibromyalgia. (A-143; Rheumatologist, Scotland).

#### Sub-theme: unhelpful pharma and the cost of keeping going

Despite different views about where care for people with fibromyalgia should be located, professionals did agree on the problematic over and misuse of medicines for the treatment of fibromyalgia-related pain. The limited benefit, side effects and potential harms associated with a dominant pharmacological approach to managing pain were emphasised by respondents. Specialist pain and rheumatology services were singled out by professionals working in general practice for initiating and perpetuating inappropriate medicines prescribing, causing problems downstream in primary care. Similarly, general practitioners were charged with escalating patients through the analgesic ladder, which some in primary care suggested was due to a lack of accessible alternative treatment options.

One issue is if they [people with fibromyalgia] attend a pain clinic and get issued Morphine and Pregabalin, the implication is the GP will continue to prescribe. […] These drugs are linked with serious problems in the long term, and it is very difficult for the GP to say you should stop them […]. They [people with fibromyalgia] view you as unsympathetic. (A-615; GP, Greater London).

We often end up prescribing pain medication because we don’t have access to other treatments. Occasionally this helps but the pain medications are not without risk of dependence, sedation [and] polypharmacy. […] We have some in-house counselling, but this is a stretched underfunded service. (A-1148; GP, Scotland).

People with fibromyalgia reported frequent use of non-NHS interventions as an alternative or adjunct to medicines, with many describing benefits such as improved well-being and function, including work ability. However, the cost of such interventions was substantial, and for some this prohibited access to care.

I paid to use a private hydrotherapy pool, but my PIP (Personal Independence Payment) was cut, so I had to cancel all the extras I was paying for. (B-368, England).

### Gaps in healthcare services

1601 NHS healthcare professionals answered the question “Are there gaps in your local healthcare service for people with fibromyalgia?”. Of these, only 182 (11.4%) said there were not any gaps and 274 (17.1%) said they did not know. The most frequently cited unmet need was a lack of available services. Over half of respondents (55.7%) stated this as an important unmet need, with 31.2% indicating that it was the most important unmet need.

Other needs frequently mentioned were lack of healthcare professionals’ knowledge or skills in assessment and treatment of fibromyalgia and long wait times to appointments. The most common reason for use of non-NHS services given by people with fibromyalgia was that services or treatments were not offered, or, if they were offered, provision was limited. Analysis of free text data relating to gaps in healthcare services led to generation of the theme ‘A low priority’.

### Theme: A low priority

Fibromyalgia is a condition of sizable impact, yet respondents across both surveys emphasised a mismatch between the needs of people with fibromyalgia in the UK and NHS provision. One general practitioner reflected that fibromyalgia ranked lower in priority than a Dupuytren’s contracture,^1^ while other respondents perceived a general lack of interest in the condition and those affected among healthcare professionals.

#### Sub-theme: A barren landscape

Some NHS healthcare professionals referred to an erosion of secondary care fibromyalgia services in their localities, leaving general practitioners and their patients with fibromyalgia in a barren landscape when the skills and resources of primary care are surpassed.

[My health board] has withdrawn their secondary care service for fibromyalgia. We have nowhere to refer these patients onto for specialist advice and support. (A-1327; GP, Wales).

In other areas where provision was present or appeared piecemeal, NHS healthcare professionals described oversubscribed services and long waiting times. Substantial gaps were reported in the availability of holistic programmes delivered by multidisciplinary teams, and psychological therapies. One general practitioner called for psychological therapists to be at the forefront of pain management approaches, while several allied health professionals believed enhancing their own psychology-based skills could go some way to bridging the shortfall in mental health support for people with fibromyalgia.

#### Sub-theme: nothing long-term

Also underpinning fibromyalgia’s low priority status were comments highlighting a lack of appropriate needs-based care provision for people with fibromyalgia throughout the life course. A lack of support outside of general practice post diagnosis for those learning to manage their fibromyalgia and during times of flare was highlighted, although one participant with fibromyalgia had worked with a general practice to address this situation.

I have …start[ed] a new patient support group. We have around 30 patient members. We have monthly meetings at [the] surgery [and] have a WhatsApp group and can meet locally for walks, coffee chat or crafts. (B-22, England).

Healthcare professionals described inadequate coverage and access to community-based opportunities such as low intensity exercise classes, wellbeing initiatives and NHS linked peer support groups. One participant living with fibromyalgia and a rheumatologist working in England outlined the impact of limited community support.

Self-management is fine in theory but really difficult to do well when in pain and exhausted. [You] need someone with expertise to coach, support and enable you to keep going. (B-270, England).

With current NHS service restrictions, once the diagnosis is made and initial treatment given, patients are discharged back to [the] GP, which should be fine but GP services [are] often really stretched and patients either don’t get the support they want or end up getting referred back to secondary care every few years to ‘query’ the diagnosis because the GP doesn’t know what to do. (A-36; Rheumatologist, South East England).

#### Sub-theme: [un]knowledgeable professionals

Professions are commonly distinguished from other occupational groups by specialist knowledge and skills [37]. Yet many participants referred to a general lack of understanding about fibromyalgia among healthcare professionals, both specialists and generalists, which was in turn perceived to link to delayed diagnosis, invalidation of people with fibromyalgia and, as in the account below, missed opportunities for interactions to support health and well-being.

I don’t find many people within the NHS… understand the condition. I feel they have opinions that are often wrong and… offensive. I go as little as possible to my doctors as they don’t understand it and therefore can’t help me. (B-274; Wales).

Stigma, commonly experienced by people with fibromyalgia, was evident in accounts from healthcare professionals, although the ‘often wrong and offensive’ opinions (outlined in the comment above) were typically attributed to other healthcare professionals rather than stated by those who participated in the survey. Occasionally, a healthcare professional did explicitly express a negative evaluation about people with fibromyalgia.

As an anaesthetist, my heart sinks when a preoperative patient announces …that they suffer with fibromyalgia, as they are often very ‘needy’ in the recovery area. Having observed and managed pain in post-operative patients for some 30 years, my impression is that the problems experienced by post-operative fibromyalgia patients are more ‘supratentorial’ in nature. (A-471; Hospital doctor, West Midlands).

Many respondents indicated a need for improved education of healthcare professionals about fibromyalgia. For example, orthoptists and midwives suggested they would benefit from increased training to support patients attending their services. However, even when professional expertise was available, it was not always used because of other service pressures.

For the last 16 years I have done a weekly fibromyalgia clinic seeing 5 new patients every week. Patients would come from across [the region]. […] However, because of a dearth of rheumatologists my fibro[myalgia] clinic has been shut on me and I now spend that clinic seeing ordinary patients who may or may not have inflammatory arthritis. Any old clinician could deal with these patients with a check list. … a lot of skill and experience is required to deal with patients with FMS [fibromyalgia syndrome] and I am one of the few rheumatologists with expertise in this field. This is an example of the NHS disrespecting people unfortunate enough to [have] FMS [fibromyalgia syndrome]. (A-1437; Rheumatologist, Wales).

## Discussion

This study offers a view of UK national health services for people with fibromyalgia from the perspective of NHS healthcare professionals. It also provides insight into the use of non-NHS services by people with fibromyalgia living in the UK. Other studies within the PACFiND research programme have investigated and reported experiences of people with fibromyalgia in relation to NHS services, in primary care specifically [35] and, more broadly, on the Healthtalk website (https://healthtalk.org/Fibromyalgia). Taken as a whole, the results of this study represent a journey across UK health services travelled by people with fibromyalgia. NHS healthcare professionals, in keeping with clinicians in other countries, report substantial variability diagnosing fibromyalgia. Of the general practitioners who responded to our survey, three out of ten reported not diagnosing fibromyalgia, influenced by low levels of diagnostic confidence and perceptions about the nature and utility of the fibromyalgia label. Rheumatologists are a key point of referral for a diagnosis of fibromyalgia, but this can delay initiation of treatment and management. Education and pharmacotherapy are the mainstay of treatment provided to people with fibromyalgia by NHS healthcare professionals, however, both patients and professionals recognise room for improvement in these approaches. Although the effectiveness of pharmacological and non-pharmacological treatments are rated similarly by people with fibromyalgia in the UK, non-pharmacological treatments have higher acceptability to those affected [38].

Referrals between NHS professionals to support people with fibromyalgia with their recovery are common, yet few professionals reported a local coordinated care path along which to direct people. Similar to health professionals’ experiences in other publicly funded health services, provision of non-pharmacological interventions for people with fibromyalgia, such as psychological therapies, is low. A lack of available NHS services, in particular multidisciplinary clinics providing holistic care and community-based opportunities linked to general practice to support self-management, was reported frequently and the primary reason people with fibromyalgia accessed non-NHS care.

While some of our results concerning diagnosis, treatment and management, and gaps in healthcare services bear out findings from previous research in the field [14, 17, 39–43], we have identified several issues less commonplace in the fibromyalgia literature. First, healthcare professionals’ possible bias about people with self-diagnosed fibromyalgia. Second, the substantial challenge facing general practitioners in the UK when seeking involvement of secondary care services for people with fibromyalgia. Third, the lack of available mental health and multidisciplinary holistic services in some regions to support people with fibromyalgia.

The findings of this study offer insights into access to healthcare for people with fibromyalgia in the UK and can be understood through the concept of candidacy. Candidacy encapsulates the processes through which a person’s eligibility for healthcare is jointly negotiated between individuals and health services [44]. These processes focus on recognition of a need for healthcare, navigation and permeability of services, presentation at services and the adjudications of healthcare professionals, offers of and resistance to care, and finally the local operating conditions through which candidacy is addressed [44]. Data from this study map to three elements of the candidacy framework in particular - presentation at services and the adjudications of healthcare professionals; permeability of services; and local operating conditions.

### Presentation at healthcare services and the adjudications of professionals

The way in which patients present to healthcare services, and the judgements made by healthcare professionals about such presentations, influences the progression of candidacy [45, 46]. Fibromyalgia is a stigmatising illness [47], shaped by societal attitudes about chronic pain and mental illness. Greater perceived stigma in people with chronic pain can heighten pain-related disability and distress [48], and affect presentation at services. Like others with invisible conditions, people with fibromyalgia commonly employ legitimacy narratives to communicate their illness, focused on symptoms and their impact, the struggle to complete everyday tasks and an inability to fulfil gender roles [49]. These narratives can be challenging to healthcare professionals in the absence of outward signs to substantiate such accounts and may lead to scepticism about symptom severity [50, 51], suspicion that people with fibromyalgia are seeking secondary gain [51, 52] or are ‘complaining women’ [52] or malingerers [53]. Such stereotypes can negatively impact patients through increased psychosocial stress [48] and healthcare professionals’ decisions about patient care [54] with potential for suboptimal management [55]. Data from interviews with healthcare professionals providing care for people with fibromyalgia suggest they may be reluctant to grant women with fibromyalgia sick leave, undertake avoidant-like behaviours to reduce contact with patients with fibromyalgia and request unnecessary investigations and treatments during interactions [50, 52]. The finding of possible bias among healthcare professionals about people with self-diagnosed fibromyalgia is salient. While the effect of self-diagnosis on professional reactions and judgements is an under researched area [56], this prejudice is a potential concern given the lack of an objective test to diagnose fibromyalgia and the prevalence of self-diagnosis among the general population [57, 58].

### Permeability of services

The substantial challenge facing general practitioners in the UK when seeking access to support from secondary care services for people with fibromyalgia is a key finding of this research. Permeability of services reflects ease of access to care by those seeking it. Services such as hospital emergency departments are highly porous, while access to specialist services necessitates a referral and at least some agreement between referrer and provider about expectations of care [59]. Our data suggests that some NHS services in the UK are impervious to people with fibromyalgia, constituted by pressure to address core individual speciality work, local access polices, and a long-standing focus in NHS services on either physical or mental health. Fibromyalgia’s low prestige ranking is also likely to play a part [60].

Rheumatologists have traditionally ‘owned’ fibromyalgia [61]. Yet, some are reluctant to accept patients with fibromyalgia for care [17, 62] and the argument for diagnosis and treatment of people with fibromyalgia outwith rheumatology services is growing [61, 63]. UK national audits show rheumatology services face challenges meeting quality standards for people with early inflammatory arthritis [64, 65] and recent guidance published by the national Getting It Right First Time (GIRFT) programme [66] recommends that care for people with fibromyalgia should be provided in primary and community settings. Going forward, it is likely that UK rheumatology services will become less permeable to people with fibromyalgia, with implications for patients and healthcare professionals, notably in general practice. Alongside this are reports that some physiotherapy services may be impenetrable to people with fibromyalgia. This is surprising given that physiotherapists are a professional group to which people with fibromyalgia are commonly referred [14, 67]. Speculatively, reduced permeability of these services could align with a discourse of self-management [68] and physiotherapists’ concern about perpetuating dependence on care [51]. Understanding more about non-physician’s views and perceptions about access to care for people with fibromyalgia may be beneficial.

Neal [42] argues that psychiatrists might take responsibility for the care of people with fibromyalgia. Around 50–70% of people with persistent physical symptoms have comorbid mental health problems [69, 70] yet some of the NHS professionals in our study reported a diagnosis of fibromyalgia is a barrier to accessing mental health services. Röhricht and Elanjithara [71] suggest that a focus on severe mental illness in secondary care mental health services means that provision for people with persistent physical symptoms in these settings is rare. A study exploring entry criteria to UK NHS adult mental health services, showed that diagnoses are inefficient proxies for risk, severity and need [72].

### Local context

Local availability of suitable NHS healthcare services is fundamental in addressing candidacy [44, 73]. Yet the lack of available multidisciplinary holistic services, psychological services, community-based services to support people in self-management and appropriately skilled staff to meet patient need, are substantial barriers to better outcomes for people with fibromyalgia and opportunities to reduce health service costs. The predominant symptom in people with fibromyalgia is persistent multisite pain [9], however, the most recent Health Survey for England undertaken in 2017 showed that only half of respondents with persistent pain and high interference in usual daily activities had seen a pain specialist [74]. Specialist pain clinics in England and Wales see around 0.4% of the total national population, but not all are multidisciplinary, and fewer than two thirds offer a pain management programme [75]. Provision of pain management programmes in Ireland is patchy with long waits for access [76], while in Scotland, programmes run in 10 of the 14 health boards [77]. Alongside specialist pain services are services for people with persistent physical symptoms (also referred to as medically unexplained symptoms (MUS)), under which people with fibromyalgia fall. However, multidisciplinary teams providing MUS services, particularly in primary care, are uncommon and provision across all sectors of the health service is inadequate for need [78].

New initiatives to fill gaps in the provision of psychological services (such as England’s improving access to psychological therapies-long term condition (IAPT-LTC) programme [70]), and policy mandates to build collaborations between health services and the voluntary, community and social enterprise (VCSE) sector [79] to ensure service coverage, may go some way to address the needs of people with fibromyalgia. However, the complexity of mental health needs, inadequate confidence and expertise in general practice to manage such complexity, limited access to secondary care mental health services because of inclusion thresholds, and demand and fiscal challenges facing the VCSE sector as a result of the COVID-19 pandemic [80, 81], means that these will only be one part of the solution.

## Study limitations

The findings of this study should be considered in the light of its strengths and weaknesses. A key strength is that survey A was widely distributed to NHS organisations and professional bodies across the UK and responses were received from a broad range of NHS professionals involved in care for people with fibromyalgia. The main weaknesses of our study include the unknown representativeness of our survey populations and self-selection bias. Convenience samples may have led to under and over coverage; people with access to the internet could respond to Survey B and the questionnaire was in English. Additionally, healthcare professionals sufficiently interested in care organisation and delivery for people with fibromyalgia may have participated. Furthermore, no IP addresses were collected, so it is possible that the survey was accessed more than once by the same user. Despite these limitations, this study provides insight into accessing fibromyalgia services in the UK from the perspective of NHS professionals and people with fibromyalgia and builds on current knowledge.

## Conclusion

Our study has highlighted problems widely across the NHS in service provision and access for people with fibromyalgia in the UK, including several issues less commonly discussed: potential bias among healthcare professionals about people with self-diagnosed fibromyalgia; challenges facing general practitioners seeking involvement of secondary care services for people with fibromyalgia; and the lack of available mental health and multidisciplinary holistic services to support those affected. Changes in services occurring as a result of the COVID-19 pandemic is likely only to have exacerbated the issues found through this research. There is a need to co-design and implement integrated services that offer people with fibromyalgia timely access to healthcare professionals with expert knowledge of fibromyalgia and a holistic management plan focussed towards long-term self-management. It will be important to consider patient choice in the mode of service delivery along with cost and scalability to ensure access for those in need of support. Developing new models of care for people with fibromyalgia in primary and community care could offer opportunities to address many of the issues presented in this study.

## Supplementary Information

**Additional file 1.**

**Additional file 2.**

**Additional file 3.**

## Data Availability

The datasets used and analysed during this study are not publicly available due to the specific nature of responses from healthcare professionals and people with fibromyalgia, but they are available from the corresponding author on reasonable request.

## Footnotes

1 Dupuytren’s contracture is a thickening of the tissues of the palm and can result in clawing of the fingers.
