# Supplementary material for: UK healthcare services for people with fibromyalgia: results from two web-based national surveys (the PACFiND study)
Source: BMC Health Serv Res. 2022 Aug 3;22:989. doi: 10.1186/s12913-022-08324-4 (PMC9347075; doi:10.1186/s12913-022-08324-4)
Supplement: Supplementary file 1 — Additional file 1. [file 12913_2022_8324_MOESM1_ESM.pdf]

Mapping NHS services for people with Fibromyalgia

This survey is intended for health professionals working in the NHS who, in the last two years, have seen patients with Fibromyalgia or with signs and symptoms suggestive of Fibromyalgia (i.e. persistent widespread pain/tenderness for at least three months, with associated symptoms such as fatigue, non-restorative sleep, impaired memory/concentration and mood disorders).

This survey is being carried out by researchers from the University of Aberdeen, the University of Oxford and King's College Hospital NHS Foundation Trust to find out about NHS services for people with Fibromyalgia. It is part of a research study called PACFiND – PAtient-centred Care for Fibromyalgia: New pathway Design, funded by Versus Arthritis, which aims to improve healthcare for people with Fibromyalgia. You can find out more about the study here.

We would like to invite you to participate in this survey which should take no more than 5 minutes of your time to complete. Please feel free to forward this survey to appropriate colleagues.

Your participation is entirely voluntary and you can withdraw at any point. You are free to omit question(s). Your responses will be anonymous and the data will be stored securely at the University of Aberdeen for 10 years. After this time it will be destroyed.

If you have any questions about the survey you can contact the PACFiND team on pacfind@abdn.ac.uk

Thank you for your time.

Consent to participate

I have read and understood the information above and consent to participate in this survey*.

◯ Yes
◯ No

Demographic information

What is your main job in healthcare?

- ◯ General practitioner
- ◯ Hospital doctor
- ◯ Nurse
- ◯ Occupational therapist
- ◯ Physiotherapist
- ◯ Psychologist
- ◯ Psychiatrist
- ◯ Mental health practitioner
- ◯ Other - please specify in pop-up box below

Other main job - please specify: ______________________________

In what specialty is your main job?

- ◯ Gastroenterology
- ◯ General Practice
- ◯ Neurology
- ◯ Pain Medicine
- ◯ Psychiatry
- ◯ Psychology
- ◯ Rehabilitation
- ◯ Rheumatology
- ◯ Other - please specify in pop-up box below

Other specialty - please specify: ______________________________

What country is your main job in?

- ◯ England
- ◯ Northern Ireland
- ◯ Scotland
- ◯ Wales

If your main job is in England, please select your region:

- ◯ South East
- ◯ South West
- ◯ London
- ◯ East of England
- ◯ East Midlands
- ◯ West Midlands
- ◯ Yorkshire and the Humber
- ◯ North East
- ◯ North West

If your main job is in Northern Ireland, please select your region:

- ◯ Belfast
- ◯ Southern
- ◯ Northern
- ◯ Western
- ◯ South Eastern

If your main job is in Scotland, please select your region:

- ◯ Ayrshire and Arran
- ◯ Borders
- ◯ Dumfries and Galloway
- ◯ Fife
- ◯ Forth Valley
- ◯ Grampian
- ◯ Greater Glasgow and Clyde
- ◯ Highlands and Western Isles
- ◯ Lanarkshire
- ◯ Lothian
- ◯ Orkney and Shetland
- ◯ Tayside

If your main job is in Wales, please select your region:

- ◯ Abertawe Bro Morgannwg
- ◯ Aneurin Bevan
- ◯ Betsi Dadwaladr
- ◯ Cardiff and Vale
- ◯ Cwm Taf
- ◯ Hywel Dda
- ◯ Powys

What is the healthcare setting for your main job?

- ◯ General practice
- ◯ Primary healthcare centre
- ◯ Acute hospital
- ◯ Community hospital
- ◯ Other - please specify in pop-up box below

Other healthcare setting - please specify: ________________________________

Which of the following best describes the main setting from which you personally deliver healthcare for patients with Fibromyalgia, or signs and symptoms suggestive of Fibromyalgia?

- ◯ Urban
- ◯ Suburban
- ◯ Rural

In addition to this setting, do you personally deliver healthcare for patients with Fibromyalgia, or signs and symptoms suggestive of Fibromyalgia in other settings?

- ◯ Yes
- ◯ No

Please select any other setting(s) from which you personally deliver healthcare:

- ☐ Urban
- ☐ Suburban
- ☐ Rural

Diagnosing Fibromyalgia

Do you diagnose Fibromyalgia*?

*Symptoms are: persistent widespread pain/tenderness with associated symptoms such as fatigue, non-restorative sleep, impaired memory/concentration and mood disorders.

◯ Yes
◯ No

What age groups do you diagnose?

◯ Adults (≥18 years)
◯ Adolescents (13 to 17 years)
◯ Adults AND Adolescents

What screening/assessment tool(s)/technique(s) do you use to diagnose Fibromyalgia? Please tick all that apply.

☐ Widespread Pain Index
☐ Symptom Severity Scale
☐ Tender Point examination
☐ Clinical opinion
☐ None
☐ Other - please specify in pop-up box below

If you use other screening/assessment tools/technique(s) - please specify:

____

Do you refer to other providers to make a diagnosis of Fibromyalgia?

◯ Yes
◯ No

To which providers do you refer? Please tick all that apply.

☐ General practitioner
☐ Occupational therapist
☐ Physician in pain medicine
☐ Physiotherapist
☐ Psychologist
☐ Psychiatrist
☐ Gastroenterologist
☐ Neurologist
☐ Rheumatologist
☐ Other - please specify in pop-up box below

Please specify which other provider(s) you refer patients to:

____

Which of these providers that you refer patients to for a diagnosis are located in your healthcare setting? Please tick all that apply.

☐ None
☐ General practitioner
☐ Occupational therapist
☐ Physician in pain medicine
☐ Physiotherapist
☐ Psychologist
☐ Psychiatrist
☐ Gastroenterologist
☐ Neurologist
☐ Rheumatologist
☐ Other - please specify in pop-up box below
☐ Don't know

Please specify which of the other provider(s) that you refer patients to for a diagnosis are located in your healthcare setting:

____

Treatment/management of patients with Fibromyalgia

Do you personally provide treatment/management* for patients with Fibromyalgia?

○ Yes
○ No

*Treatment/management is considered any intervention that aims to combat Fibromyalgia or alleviate signs and symptoms suggestive of Fibromyalgia.

Treatment/management interventions

What treatment/management interventions do you personally provide for patients with Fibromyalgia? Please tick all that apply.

- ☐ Information leaflet
- ☐ Education (e.g. about sleep hygiene, exercise or mental health)
- ☐ Structured land-based exercise (e.g. physiotherapist-led fitness class)
- ☐ Structured water-based exercise (e.g. hydrotherapy)
- ☐ A medicines prescription
- ☐ A recommendation for a medicine or an over the counter medicine
- ☐ Manual therapies (e.g. massage, manipulation)
- ☐ Acupuncture
- ☐ Psychological therapies (e.g. CBT, counselling)
- ☐ A multicomponent programme (e.g. a pain management programme)
- ☐ Other - please specify in pop-up box below

Please specify what other treatment/management interventions you provide:

____

Multicomponent programmes

What intervention(s) are delivered within your multicomponent programme? Please tick all that apply.

- ☐ Exercise therapy
- ☐ Meditative movement (e.g. Tai Chi, Yoga)
- ☐ Psychological Therapy (e.g. CBT)
- ☐ Sleep hygiene / management
- ☐ Education and advice
- ☐ Other - please specify in pop-up box below

Please specify what other multicomponent intervention(s) you provide:

____

How is your multicomponent programme delivered? Please tick all that apply.

- ☐ One to one face-to-face sessions
- ☐ Group-based face-to-face sessions
- ☐ Telephone sessions/support
- ☐ Online sessions/support
- ☐ Other - please specify in pop-up box below

Please specify what other way(s) your multicomponent programme is delivered:

____

What is the overall length of your multicomponent programme? Please enter a number of weeks.

____

How many sessions are there within your multicomponent programme?

____

What is the length of each session? Please enter a number of minutes.

____

Where is your multicomponent programme delivered? Please tick all that apply.

- ☐ General practice surgery
- ☐ Hospital setting
- ☐ Home
- ☐ Leisure centre
- ☐ Community venue (e.g. community centre)
- ☐ Other - please specify in pop-up box below

Please specify the other location(s) where your multicomponent programme is delivered:

____

Which healthcare workers deliver this multicomponent programme? Please tick all that apply.

- ☐ Doctors
- ☐ Psychologists
- ☐ Nurses
- ☐ Physiotherapists
- ☐ Occupational Therapists
- ☐ Mental health practitioners
- ☐ Pharmacists
- ☐ Assistant practitioners, e.g. physiotherapy assistant
- ☐ Other - please specify in pop-up box below

Please specify which other healthcare workers deliver this multicomponent programme?

____

Referring to other providers for treatment/management

Do you refer patients with Fibromyalgia to other providers for treatment/management?

- ◯ Yes
- ◯ No

To which providers do you refer for treatment/management? Please tick all that apply.

- ☐ General practitioner
- ☐ Psychologist
- ☐ Nurse
- ☐ Physiotherapist
- ☐ Assistant practitioner, e.g. physiotherapy assistant, health trainer
- ☐ Occupational Therapist
- ☐ Mental health practitioner
- ☐ Pharmacist
- ☐ Physician in pain medicine
- ☐ Psychiatrist
- ☐ Rheumatologist
- ☐ Other - please specify in pop-up box below

Which other provider(s) do you refer to for treatment/management? Please specify.

____

Which of these providers that you refer to for treatment/management are located in your healthcare setting? Please tick all that apply

- ☐ None
- ☐ General practitioner
- ☐ Psychologist
- ☐ Nurse
- ☐ Physiotherapist
- ☐ Assistant practitioner, e.g. physiotherapy assistant, health trainer
- ☐ Occupational Therapist
- ☐ Mental health practitioner
- ☐ Pharmacist
- ☐ Physician in pain medicine
- ☐ Psychiatrist
- ☐ Rheumatologist
- ☐ Other - please specify in pop-up box below
- ☐ Don't know

Please specify which of the other provider(s) that you refer patients to for treatment/management are located in your healthcare setting:

____

Signposting

Do you direct or signpost patients with Fibromyalgia to other services or interventions?

◯ Yes
◯ No

What services or interventions do you signpost patients with Fibromyalgia to? Please tick all that apply.

☐ Self-help book(s) / leaflets
☐ On-line resources
☐ Support group(s) and or workshops
☐ Other - please specify in pop-up box below

What other services or interventions do you signpost patients with Fibromyalgia to? Please specify:

____

Gaps in service provision

In your opinion, are there gaps in your local healthcare services for patients with Fibromyalgia?

◯ Yes
◯ No
◯ Don't know

What do you consider is the most important unmet need for the care of patients with Fibromyalgia in your locality?

◯ Lack of available services (e.g. physiotherapy, multicomponent programmes)
◯ Limited transport availability between patient and provider
◯ Long wait times to appointments
◯ Lack of health professional's knowledge and skills in assessment, treatment/management of patients with Fibromyalgia
◯ Lack of adequate time during appointments
◯ Restrictive service delivery policies
◯ Funding issues (e.g. underfunding, high treatment costs)
◯ Continuity of relations between provider and patient
◯ Lack of access to shared patient medical records
◯ Limited communication and coordination between providers
◯ Other (please specify)

Please specify what other most important unmet need exists in your locality:

____

Please select other important unmet needs for the care of patients with Fibromyalgia in your locality

☐ Lack of available services (e.g. physiotherapy, multicomponent programmes)
☐ Limited transport availability between patient and provider
☐ Long wait times to appointments
☐ Lack of health professional's knowledge and skills in assessment, treatment/management of patients with Fibromyalgia
☐ Lack of adequate time during appointments
☐ Restrictive service delivery policies
☐ Funding issues (e.g. underfunding, high treatment costs)
☐ Continuity of relations between provider and patient
☐ Lack of access to shared patient medical records
☐ Limited communication and coordination between providers
☐ Other - please specify in pop-up box below

Please specify what other important unmet needs there are in your locality:

____

If you have any further comments about service gaps in your locality, we would be grateful if you would include them here.

____

Other comments

If you have any other comments about any aspect of this survey we would be grateful if you would include them here. ________________________________

Contact details

It would be most helpful if you would provide the address of your service / practice. This will help us to identify potential case study sites for future research associated with PACFiND.

____

Postcode of service/practice:

____

Thank you

Thank you for completing this survey. Please remember to press the submit button below!
