# Supplementary material for: UK healthcare services for people with fibromyalgia: results from two web-based national surveys (the PACFiND study)
Source: BMC Health Serv Res. 2022 Aug 3;22:989. doi: 10.1186/s12913-022-08324-4 (PMC9347075; doi:10.1186/s12913-022-08324-4)
Supplement: Supplementary file 2 — Additional file 2. [file 12913_2022_8324_MOESM2_ESM.pdf]

Non-NHS services used by people with Fibromyalgia

Online information and consent form

About this survey

The purpose of this survey is to find out about non-NHS services and treatments that people with Fibromyalgia use to help manage their condition. Non-NHS services include, but are not limited to, healthcare delivered by private organisations/professionals, voluntary and community organisations. You may pay for some of these non-NHS services. Your responses will help us understand what services outside the NHS people with Fibromyalgia living in the UK currently use.

This survey is part of a research study called PACFiND – PAtient-centred Care for Fibromyalgia: New pathway Design conducted by researchers at the University of Aberdeen, the University of Oxford and King's College Hospital NHS Foundation Trust. The research is funded by Versus Arthritis and aims to improve healthcare for people with Fibromyalgia. You can find out more here.

Who can take part in this survey?

This survey is for people aged 16 years or older with diagnosed Fibromyalgia living in the UK. We kindly request that you do not complete the questionnaire if you are aged under 16 or if you do not have Fibromyalgia.

What does it involve and how long will it take?

The survey has seven questions (including multiple choice and text based answers) and it will need to be done in one go. It should take no more than 5 minutes of your time to complete.

Are there any risks?

We believe there are no known risks associated with undertaking this survey. Your participation is entirely voluntary and you can stop completing the survey at any point. If you do not wish to answer a question, you can leave it blank. The ethical aspects of this study been approved by the Life Sciences and Medicine Ethics Review Board at the University of Aberdeen.

What about privacy and confidentiality?

Responses to the survey will be anonymous and will be stored securely on a password protected computer at the University of Aberdeen for 10 years. After this time, the data will be destroyed. Only the researcher conducting this survey and the PACFiND study coordinator will have access to the data. The results of the survey may be published but will not include identifiable information.

Can I obtain a summary of the survey findings?

Yes, a short summary of the findings of this survey will be available on the PACFiND website once the study has been

completed.

What if I have a question?

If you have any questions about the survey, you can contact the PACFiND research study team on pacfind@abdn.ac.uk or by phone on 01224 43 7087.

What do I do now?

If you have read and understood the above information and would like to participate in the survey, please continue and confirm your eligibility below. If you do not wish to participate further, please exit this web page and we thank you for your time.

Eligibility criteria

This survey is designed to be completed only by people who are aged 16 or older. We kindly request that you do not complete this survey if you are under 16 years of age.

Are you aged 16 or older?

◯ Yes
◯ No

Consent to participate

I have read and understood the information above and consent to participate in this survey.

◯ Yes
◯ No

Demographic information

What country do you live in?

- ◯ England
- ◯ Northern Ireland
- ◯ Scotland
- ◯ Wales

What region do you live in?

- ◯ South East
- ◯ South West
- ◯ London
- ◯ East of England
- ◯ East Midlands
- ◯ West Midlands
- ◯ Yorkshire and the Humber
- ◯ North East
- ◯ North West

What region do you live in?

- ◯ Belfast
- ◯ Southern
- ◯ Northern
- ◯ Western
- ◯ South Eastern

What region do you live in?

- ◯ Ayrshire and Arran
- ◯ Borders
- ◯ Dumfries and Galloway
- ◯ Fife
- ◯ Forth Valley
- ◯ Grampian
- ◯ Greater Glasgow and Clyde
- ◯ Highlands and Western Isles
- ◯ Lanarkshire
- ◯ Lothian
- ◯ Orkney and Shetland
- ◯ Tayside

What region do you live in?

- ◯ Abertawe Bro Morgannwg
- ◯ Aneurin Bevan
- ◯ Betsi Dadwaladr
- ◯ Cardiff and Vale
- ◯ Cwm Taf
- ◯ Hywel Dda
- ◯ Powys

Use of non-NHS services

In the past 12 months, have you used non-NHS services to help you manage your Fibromyalgia?

Non-NHS services include, but are not limited to: Healthcare delivered by private organisations/providers and /or voluntary and community organisations. You may pay for some of these non-NHS services.

◯ Yes
◯ No

Please specify what non-NHS funded services/treatments you have accessed in the past 12 months (please tick all that apply).

☐ Alexander technique
☐ Acupuncture
☐ Aromatherapy
☐ Chiropractic
☐ Massage therapy
☐ Herbal medicine
☐ Homeopathy
☐ Hypnotherapy
☐ Meditation
☐ Mindfulness
☐ Osteopathy
☐ Physiotherapy (non NHS)
☐ Pilates, Tai Chi, Yoga
☐ Naturopathy
☐ Nutritional therapy
☐ Reflexology
☐ Reiki
☐ Support group(s)
☐ Workshop (e.g. sleep or job support)
☐ Other

You have indicated that you have used other non-NHS services/treatments within the past 12 months. Please specify:

____

Frequency of treatment

How many times over the past 12 months did you use this service/treatment?

If you ticked more than one service/treatment, please tell us about the one you used the most in the past 12 months.

- ◯ About once in the past 12 months
- ◯ About two or three times in the past 12 months
- ◯ About four to six times in the past 12 months
- ◯ About once or twice per month
- ◯ About once or twice per week
- ◯ More than twice a week
- ◯ Don't know

Reasons for use of non-NHS services

Could you please tell us why you accessed this service/treatment? Please tick all that apply.

- ☐ Unsatisfactory NHS waiting times
- ☐ Lack of flexibility of NHS appointments
- ☐ Service/treatment not offered by the NHS or limited provision
- ☐ Perceived lack of effectiveness of NHS treatment
- ☐ Other
- ☐ Don't know

You have indicated other reasons for accessing non-NHS services within the past 12 months. Please specify: ______________________________

Experiences of non-NHS services

Could you please tell us about your experience(s) of using this service/ treatment?

If you accessed more than one service/treatment, please tell us about the one you used the most in the past 12 months.

____

To help us further with the PACFiND research study, it would be most helpful if you would provide the address of the main non-NHS organisation/provider that you have used in the past 12 months. We will only use this information to help us choose areas of the country for further research associated with the PACFiND study.

____

Postcode of non-NHS organisation/provider: ____________________________

Other comments

If you have any other comments about any aspect of this survey we would be grateful if you would include them here. ____

Thank you

Thank you for completing this survey. Please remember to press the submit button below!
