# Supplementary material for: UK healthcare services for people with fibromyalgia: results from two web-based national surveys (the PACFiND study)
Source: BMC Health Serv Res. 2022 Aug 3;22:989. doi: 10.1186/s12913-022-08324-4 (PMC9347075; doi:10.1186/s12913-022-08324-4)
Supplement: Supplementary file 3 — Additional file 3. [file 12913_2022_8324_MOESM3_ESM.docx]

**Checklist for Reporting Results of Internet E-Surveys (CHERRIES)**

| **Item category** | **Checklist item** | **Explanation** |
| --- | --- | --- |
| Design |  | Two cross-sectional surveys.  Survey A: The target population was NHS healthcare professionals consulting with people with fibromyalgia or signs and symptoms suggestive of fibromyalgia, within the last two years.  Survey B: The target population was people aged 16 years or older living in the UK with a diagnosis of fibromyalgia and using non-NHS services to help them self-manage their condition.  Convenience sampling. |
| Ethics | Ethical approval | Survey A: The Health Research Authority (HRA) confirmed survey A was not considered to be research and did not require review by an NHS Research Ethics Committee. Survey A was registered as a service evaluation with NHS Grampian (Project ID 4679).  Survey B: Approved by the University of Aberdeen School of Medicine, Medical Sciences and Nutrition Ethics Review Board (previously the University of Aberdeen College Ethics Review Board) (CERB/2019/11/1805). |
|  | Informed consent | Consent to participate was obtained. Potential participants were informed about the purpose of the surveys, the research investigators, and the length of the survey. Potential participants were advised they could stop completing the survey at any point. Respondents to Survey B were asked to confirm they were aged 16 years or older. |
|  | Data protection | Participants were informed that the data would be stored on secure servers at the University of Aberdeen for 10 years and that the results of the survey may be published but would not include any identifiable information. |
| Development and pre-testing |  | The survey instruments were developed by drawing on literature in the field. Piloting was undertaken to test out the questions and procedures, including questionnaire usability and technical functionality. |
| Recruitment process | Open/ closed survey | Both surveys were open. |
|  | Contact mode | Survey A: The link to the survey was embedded in an email invitation to organisations and professional associations. |
|  | Advertising the survey | Survey A was advertised in organisational newsletters, circulars and on web sites.  Survey B was advertised on the websites and social media channels of Versus Arthritis and Fibromyalgia Action UK and on the University of Aberdeen-hosted PACFiND website.  The wording of the announcements can be found below. |
| Survey administration | Web / Email | Survey A: Email  Survey B: Web  Responses were captured through the REDCap online survey platform and stored on secure servers at the University of Aberdeen. |
|  | Context | Survey A was sent to NHS and professional organisations, inviting them to distribute the survey to NHS staff.  Survey B was posted on social media by Versus Arthritis and Fibromyalgia Action UK and both surveys were advertised through the twitter feeds of the PACFiND project and investigators. |
|  | Mandatory / voluntary | Voluntary. |
|  | Incentives | No incentives were offered. |
|  | Time/Date | Survey A responses were collected between 11th September 2019 to 5^th^ January 2020.  Survey B responses were collected between 13^th^ January to 3^rd^ February 2020. |
|  | Randomisation of items | No |
|  | Adaptive questioning | Adaptive questioning was used, with participants offered relevant questions based on their previous responses. |
|  | Number of items | Survey A was comprised of 27 items. Survey B comprised 9 items. |
|  | Number of screens | Survey A was distributed over 13 screens.  Survey B was distributed over 10 screens. |
|  | Completeness checks | Participants could choose to omit questions. No assessment of the number of completed items was undertaken. |
|  | Review step | A back button was available for participants to change their responses during survey completion if necessary. The surveys had to be completed in one sitting. |
| Response rate | Unique site visitor | Not determined. |
|  | View rate | Not applicable. |
|  | Participation rate | Not applicable. |
|  | Completion rate | Survey A: Of 2079 responses, 1701 provided data about the organisation and delivery of healthcare for people with fibromyalgia, giving a completion rate of 81.8%.  Survey B: Of 875 responses, 549 provided data about the use of non-NHS services by people with fibromyalgia living in the UK, giving a completion rate of 62.7%. |
| Preventing multiple entries | Cookies used | No |
|  | IP check | IP addresses were not gathered. |
|  | Log file analysis | No |
|  | Registration | Survey A – entry was via 15 links provided to different regions and organisations. |
| Analysis | Handling of incomplete surveys | Complete and incomplete surveys were included provided information about the organisation, delivery and use of healthcare services was provided. |
|  | Questionnaires submitted with atypical timestamps | Not applicable. |
|  | Statistical correction | Not applicable. |

**Survey Announcements**

**Survey A**

**For bulletin or newsletter**:

**Headline:** Can you help us improve healthcare for people with fibromyalgia in the UK?

**Main text:** PACFiND is a project aimed at developing new pathways of care for people with fibromyalgia ([www.abdn.ac.uk/pacfind](http://www.abdn.ac.uk/pacfind) ). We are looking for healthcare professionals who see patients with fibromyalgia to take part in a short online survey. This will help us understand current provision of NHS services for people with fibromyalgia. Please click **here** to complete this survey.

**For direct emails**:

**Email Subject line:** UK survey of NHS services for people with Fibromyalgia

**Email Main text**:

Dear colleague

I am writing to request your participation in a brief survey. The survey is part of a project called PACFiND - PAtient-centred Care for Fibromyalgia: New pathway Design, funded by Versus Arthritis, which aims to improve healthcare for people with Fibromyalgia. Your responses to this survey will help us to understand current provision of NHS services for people with Fibromyalgia in the UK.

This survey is intended for health professionals who, in the last two years, have seen patients with Fibromyalgia or with signs and symptoms suggestive of Fibromyalgia (i.e. persistent widespread pain/tenderness for at least three months, with associated presentations, such as fatigue, non-restorative sleep, impaired memory/concentration and mood disorders).

Healthcare providers in England, Northern Ireland, Scotland and Wales are invited to complete the survey which should take no more than 5 minutes of your time. Your participation is entirely voluntary and your responses will be anonymous.

Please click **here** or copy and paste the link below into your Internet browser to go to the survey.

<https://redcap.abdn.ac.uk/surveys/?s=HX9DCP8ACA>

If you would prefer, you can access the survey via the PACFiND website: <https://www.abdn.ac.uk/iahs/research/epidemiology/survey-for-healthcare-professionals-1607.php>

If you have any comments or questions, please contact the PACFiND team on pacfind@abdn.ac.uk

Thank you for your time.

Professor Gary Macfarlane on behalf of the PACFiND study team

<https://www.abdn.ac.uk/pacfind>

**Survey B**

**For web platforms**:

**Subject line:** Can you help us find out about non-NHS services that people with Fibromyalgia use to help manage their condition?

**Main text:** Researchers from the University of Aberdeen, the University of Oxford and King’s College Hospital NHS Foundation Trust want to find out what non-NHS services people diagnosed with Fibromyalgia living in the UK use to help manage their condition. Can you help? Please click **here** to complete this short survey. Thank you!

**For Twitter:**

Calling UK people living with #Fibromyalgia. We are trying to find out about services outside the NHS that people with Fibromyalgia use to help manage their condition. Can you help? Short survey **here**. #fibro @PACFiND @fmauk @VersusArthritis
